# PyAGH: a python package to fast construct kinship matrices based on different levels of omic data

**DOI:** 10.1186/s12859-023-05280-6

**Published:** 2023-04-18

**Authors:** Wei Zhao, Qamar Raza Qadri, Zhenyang Zhang, Zhen Wang, Yuchun Pan, Qishan Wang, Zhe Zhang

**Affiliations:** 1 Department of Animal Science, College of Animal Sciences, Zhejiang University, 866# Yuhangtang Road, Hangzhou, 310058 China; 2 Department of Animal Science, School of Agriculture and Biology, Shanghai Jiao Tong University, 800# Dongchuan Road, Shanghai, China; 3 Hainan Research Institute, Zhejiang University, 11# Yonyou Industrial Park, Yazhou Bay Science and Technology City, Sanya, 572025 China

**Keywords:** Kinship matrices, Python package, Omic data

## Abstract

**Background:**

Construction of kinship matrices among individuals is an important step for both association studies and prediction studies based on different levels of omic data. Methods for constructing kinship matrices are becoming diverse and different methods have their specific appropriate scenes. However, software that can comprehensively calculate kinship matrices for a variety of scenarios is still in an urgent demand.

**Results:**

In this study, we developed an efficient and user-friendly python module, PyAGH, that can accomplish (1) conventional additive kinship matrces construction based on pedigree, genotypes, abundance data from transcriptome or microbiome; (2) genomic kinship matrices construction in combined population; (3) dominant and epistatic effects kinship matrices construction; (4) pedigree selection, tracing, detection and visualization; (5) visualization of cluster, heatmap and PCA analysis based on kinship matrices. The output from PyAGH can be easily integrated in other mainstream software based on users’ purposes. Compared with other softwares, PyAGH integrates multiple methods for calculating the kinship matrix and has advantages in terms of speed and data size compared to other software. PyAGH is developed in python and C +  + and can be easily installed by pip tool. Installation instructions and a manual document can be freely available from https://github.com/zhaow-01/PyAGH.

**Conclusion:**

PyAGH is a fast and user-friendly Python package for calculating kinship matrices using pedigree, genotype, microbiome and transcriptome data as well as processing, analyzing and visualizing data and results. This package makes it easier to perform predictions and association studies processes based on different levels of omic data.

## Background

Kinship matrix, a symmetrical matrix representing the pairwise relatedness between individuals, was initially proposed to account for variance–covariance structure of breeding values (additive genetic effects) implemented in the best linear unbiased prediction (BLUP) method using pedigree information. With the development of high throughput genotyping methods during the last two decades, the kinship matrices were calculated using genome-wide markers and can be used to account for cryptic relatedness between pairwise individuals in genome wide association studies (GWAS) and to fit polygenicity in genomic prediction (GP), such as genomic best linear unbiased prediction (GBLUP) method [1, 2]. Therefore, how to construct kinship matrix is an important factor to control false discoveries in GWAS and obtain high accuracy in GP for different traits or diseases.

Actually, methods for constructing kinship matrices are becoming diverse and different methods have their specific appropriate application scenarios. For instance, under the occasion that only part of individuals are genotyped in the population, the kinship matrix can be calculated in combination of pedigree and genotypes, which can be further implemented in GP [3] and GWAS [4], which are well known as single-step methods in animal and plant breeding area. Meanwhile, when GWAS or GP is applied in a large cohort composed of multiple populations, Wientjes et al. (2017) suggested to construct a kinship matrix considering the heterogenous minor alle frequencies (MAF) across different populations. And the results obtained by simulated data showed that when the across-population genomic relationships ﻿were scaled by the within-population allele frequency, the genetic correlation was estimated unbiasedly. In addition, nonadditive effects, including the interaction between the effects of alleles either at the same locus (dominance) or between the allels of multiple genetic loci (epistasis), contributes significantly to phenotypic variation associated with the expression of polygenic complex traits [5]. Nonadditive effects are considered as a possible explanation for the "missing heritability", that is, marginal genetic effects that cannot be accounted for in GWAS or GP. Some studies have shown that considering nonadditive effects can improve the accuracy of predictions [6, 7]. However, the mainstream GWAS and GP software such as GCTA [8] or DMU [9] either fail to calculate such extended kinship matrices or can’t output these matrices, which limits its further application.

Furthermore, due to the development of multi-omics, kinship matrix can be calculated not only at the genomic level, but actually at multi-omic level. The concept of kinship should therefore be extended to improve its application in association and prediction studies. For instance, prediction of phenotypes based on transcriptome [10] or microbiome [11] improves the accuracy by utilizing more data. Microbiome-wide association studies [12] and transcriptome association studies [13] can further explore the mechanism of different omics on polygenic complex traits. However, there is no software available to meet such a need to calculate kinship matrices based on abundance data from transcriptome or microbiome.

Therefore, we developed the PyAGH package to calculate kinship matrices using a variety of methods based on different levels of omics data for different application scenarios. PyAGH can calculate additive, dominant and epistatic kinship matrices based on genomic data within one population and different additive kinship matrices across multiple populations efficiently. It also supports construction of kinship matrices using pedigree, microbiome and transcriptome data. In addition, the output of PyAGH can be easily provided to downstream mainstream software, such as DMU [9], GCTA [8], GEMMA [14] and BOLT-LMM [15]. Thus, these user-friendly features allow novice users to focus on the analysis rather than technical aspects of installation and execution.

### Implementation

The PyAGH package is implemented in Python programming language. It contains multiple Python3 and C +  + scripts with all the functions required for the program to execute. Some functions are written in C +  + via pybind11 (https://github.com/pybind/pybind11) to accelerate the computation speed. To be able to handle high-density genomic data, PyAGH supports multi-threaded computation, as well as split-chromosome computation based on the chunk matrix theory. In addition, for large-scale pedigree data, sparse matrices are used to save memory as well as to increase speed through multithreading. PyAGH has been successfully tested on machines running Unix-based operation system (OS) (macOS/Linux) and Windows. A detailed description of all algorithms and functions of PyAGH is provided in the user manual available at https://github.com/zhaow-01/PyAGH. Basic information of PyAGH’s functions has been summarized in Table 1.

**Table 1:** Summary of PyAGH’s functions

Category	Function	Description
Pedigree	sortPed()	Sort the pedigree data according to the correct birth date of individuals and check for various errors in the pedigree like offspring born before its parents, same offspring have different parents, loop in pedigree and etc
	selectPed()	Select pedigree based on specific individuals and generations
	makeA()	Construct kinship matrix based on pedigree information for additive effect. Option to use sparse matrix for memory saving
	makeD()	Construct kinship matrix based on pedigree information for dominant effect. Option to use multithreading when there are multiple CPU
Genome	makeG()	Construct kinship matrix based on genotype data for additive effect. Option to use different methods
	makeG_inter()	Construct kinship matrix based on genotype data for dominance and epistatic effect. Option to use multithreading
	makeH()	Combine information of pedigree and genotypes to construct kinship matrix for both genotyped and ungenotyped individuals
Microbiome	makeM()	Construct kinship matrix based on microbiome data
Transcriptome	makeT()	Construct kinship matrix based on transcriptome data
Composition analysis and visualization	coefKinship()	Calculate the ancestry coefficients using kinship matrix
	coefInbreeding()	Calculate the inbreeding coefficients using kinship matrix
	cluster()	Cluster analysis of the kinship matrix and plot the result
	pca()	Principal component analysis of kinship matrix and plot the result
	heat()	Plot the heatmap of a kinship matrix
	gragh()	Plot family tree tracing back up to three generations of an individual

### Construction of kinship matrix based on pedigree data

Kinship matrix is the core of traditional breeding in best linear unbiased prediction (BLUP) which is a famous classical method and widely used in breeding since 1950s [16]. *makeA() and makeD()* are the functions in PyAGH used to construct kinship matrices based on pedigree information for additive and dominant effects, respectively. The additive effect refers to the cumulative effect between alleles and non-alleles, which is a fixed component of intergenerational inheritance and is also called breeding value in breeding. The dominant effect refers to the difference between the effect value of each gene and its additive effect value, which is derived from the effect of the interaction between alleles and is a non-additive effect, called dominant deviation. This effect can be inherited but not fixed and is the main part of the heterozygous advantage. The program for function *makeA()* improves the speed of computation by referring to the algorithm propose by Meuwissen and Luo [17]. And *makeD()* further calculates the dominant effects on the basis of *makeA().* To improve the speed of computation, both functions were written in C +  + and support multi-threaded operation, while using sparse matrices to save memory.

### Construction of kinship matrix based on genomic data

With the development of genotyping technology, more and more genomic data are available for GP, like GBLUP method [18]. The *makeG()* function provides four methods for calculating the additive effects kinship matrix. In a single population, the first method to calculate G matrix was developed by VanRaden [1]:

1 $$\begin{array}{c}G=\frac{\sum \left({x}_{ij}-2{p}_{i}\right)\left({x}_{ik}-2{p}_{i}\right)}{\sum 2{p}_{i}\left(1-{p}_{i}\right)}\end{array}$$

where ${x}_{ij}$ and ${x}_{ik}$ are the genotypes of the *i*th marker in individuals *j* and *k* (denoted as 0, 1 and 2). ${p}_{i}$ is the minor allele frequency (MAF) of the *i*th marker. The second method of calculating G matrix was developed by Yang et al. [2]:

2 $$\begin{array}{c}G=\frac{1}{m}\times \sum \frac{\left({x}_{ij}-2{p}_{i}\right)\left({x}_{ik}-2{p}_{i}\right)}{2{p}_{i}\left(1-{p}_{i}\right)}\end{array}$$

where *m* is the number of markers, while other symbols represent the same meaning as formula (1). When computing G matrix in a combined popualtion, due to the differences in MAF between different populations, the direct use of the above method may bring bias. PyAGH provides two alternative methods to consider the heterogeneity of genetic structure of the combined population. One method for calculationg G matrix considering MAF differences between populations was developed by Chen et al. [19]:

3 $$\begin{array}{c}G=\left[\begin{array}{cc}{\mathbf{G}}_{11}& {\mathbf{G}}_{12}\\ {\mathbf{G}}_{21}& {\mathbf{G}}_{22}\end{array}\right]=\left[\begin{array}{cc}\frac{{\mathbf{W}}_{1}{\mathbf{W}}_{1}^{\mathrm{T}}}{\sum 2{\mathrm{p}}_{1\mathrm{j}}\left(1-{\mathrm{p}}_{1\mathrm{j}}\right)}& \frac{{\mathbf{W}}_{1}{\mathbf{W}}_{2}^{\mathrm{T}}}{\sum 2\sqrt{{\mathrm{p}}_{1\mathrm{j}}\left(1-{\mathrm{p}}_{1\mathrm{j}}\right){\mathrm{p}}_{2\mathrm{j}}\left(1-{\mathrm{p}}_{2\mathrm{j}}\right)}}\\ \frac{{\mathbf{W}}_{2}{\mathbf{W}}_{1}^{\mathrm{T}}}{\sum 2\sqrt{{\mathrm{p}}_{1\mathrm{j}}\left(1-{\mathrm{p}}_{1\mathrm{j}}\right){\mathrm{p}}_{2\mathrm{j}}\left(1-{\mathrm{p}}_{2\mathrm{j}}\right)}}& \frac{{\mathbf{W}}_{2}{\mathbf{W}}_{2}^{\mathrm{T}}}{\sum 2{\mathrm{p}}_{2\mathrm{j}}\left(1-{\mathrm{p}}_{2\mathrm{j}}\right)}\end{array}\right]\end{array}$$

where ${\mathbf{G}}_{11}$ and ${\mathbf{G}}_{22}$ represent the genomic kinship matrix of individuals in two independent populations, respectively. ${\mathbf{G}}_{12}$ and ${\mathbf{G}}_{21}$ represent the genomic kinship matrix of individuals cross two populations. ${\mathbf{W}}_{1}$ and ${\mathbf{W}}_{2}$ are standardized genotypes of individuals in two populations, respectively. ${\mathrm{p}}_{1\mathrm{j}}$ and ${\mathrm{p}}_{2\mathrm{j}}$ are the minor allele frequencies of the *j*th marker calculated based on population 1 and population 2, respectively. Another method calculationg G matrix in combined populations was developed by Wientjes et al. [20]:

4 $$\begin{array}{c}G=\left[\begin{array}{cc}{\mathbf{G}}_{11}& {\mathbf{G}}_{12}\\ {\mathbf{G}}_{21}& {\mathbf{G}}_{22}\end{array}\right]=\left[\begin{array}{cc}\frac{{\mathbf{W}}_{1}{\mathbf{W}}_{1}^{\mathrm{T}}}{\sum 2{\mathrm{p}}_{1\mathrm{j}}\left(1-{\mathrm{p}}_{1\mathrm{j}}\right)}& \frac{{\mathbf{W}}_{1}{\mathbf{W}}_{2}^{\mathrm{T}}}{\sqrt{\sum 2{\mathrm{p}}_{1\mathrm{j}}\left(1-{\mathrm{p}}_{1\mathrm{j}}\right)}\sqrt{\sum 2{\mathrm{p}}_{2\mathrm{j}}\left(1-{\mathrm{p}}_{2\mathrm{j}}\right)}}\\ \frac{{\mathbf{W}}_{2}{\mathbf{W}}_{1}^{\mathrm{T}}}{\sqrt{\sum 2{\mathrm{p}}_{1\mathrm{j}}\left(1-{\mathrm{p}}_{1\mathrm{j}}\right)}\sqrt{\sum 2{\mathrm{p}}_{2\mathrm{j}}\left(1-{\mathrm{p}}_{2\mathrm{j}}\right)}}& \frac{{\mathbf{W}}_{2}{\mathbf{W}}_{2}^{\mathrm{T}}}{\sum 2{\mathrm{p}}_{2\mathrm{j}}\left(1-{\mathrm{p}}_{2\mathrm{j}}\right)}\end{array}\right]\#\end{array}$$

where the symbols represent the same meaning as formula (3).

The *makeG_inter()* function calculates the dominant effect and epistatic effect kinship matrix based on genomic data according to algorithms proposed by Xu [21]. Epistatic effects refer to the effects of interactions between non-allelic genes at different loci, where one pair of genes suppresses or masks the other pair of genes. The formulas for dominant kinship matrix (d) and four epistatic kinship matrices (aa, dd, ad, da) are show in Table 2, where the **Z** and **W** represent the genotype matrices of different coding modes. For *k*th marker in individual *j*:

**Table 2:** Formulas used to calculate marker generated kinship matrices

Type of effect	Original kinship matrix	Kinship matrix
Dominance(d)	${K}_{d}^{*}=\sum_{k=1}^{m}{W}_{k}{W}_{k}^{T}$	${K}_{d}=(\frac{1}{mean[diag\left({K}_{d}^{*}\right)]}) {K}_{d}^{*}$
Additive × additive (aa)	${K}_{aa}^{*}=\sum_{k=1}^{m-1}\sum_{{k}^{^{\prime}}=k+1}^{m}({Z}_{k}\#{Z}_{{k}^{^{\prime}}}){({Z}_{k}\#{Z}_{{k}^{^{\prime}}})}^{T}$	${K}_{aa}=(\frac{1}{mean[diag\left({K}_{aa}^{*}\right)]}) {K}_{aa}^{*}$
Dominance × dominance (dd)	${K}_{dd}^{*}=\sum_{k=1}^{m-1}\sum_{{k}^{^{\prime}}=k+1}^{m}({W}_{k}\#{W}_{{k}^{^{\prime}}}){({W}_{k}\#{W}_{{k}^{^{\prime}}})}^{T}$	${K}_{dd}=(\frac{1}{mean[diag\left({K}_{dd}^{*}\right)]}) {K}_{dd}^{*}$
Additive × dominance (ad)	${K}_{ad}^{*}=\sum_{k=1}^{m-1}\sum_{{k}^{^{\prime}}=k+1}^{m}({Z}_{k}\#{W}_{{k}^{^{\prime}}}){({Z}_{k}\#{W}_{{k}^{^{\prime}}})}^{T}$	${K}_{ad}=(\frac{1}{mean[diag\left({K}_{ad}^{*}\right)]}) {K}_{ad}^{*}$
Dominance × additive (da)	${K}_{dd}^{*}=\sum_{k=1}^{m-1}\sum_{{k}^{^{\prime}}=k+1}^{m}({W}_{k}\#{Z}_{{k}^{^{\prime}}}){({W}_{k}\#{Z}_{{k}^{^{\prime}}})}^{T}$	${K}_{da}=(\frac{1}{mean[diag\left({K}_{da}^{*}\right)]}) {K}_{da}^{*}$

5 $$\begin{array}{c}{Z}_{jk}=\left\{\begin{array}{l}+1\quad for\; A\\ 0\quad\;\; \;for H\\ -1\quad for\; B\end{array}\right. {W}_{jk}=\left\{\begin{array}{c}0\quad for\; A\\ 1\quad for\; H\\ 0\quad for\; B\end{array}\right.\end{array}$$

${Z}_{jk}$ and ${W}_{jk}$ represent the codes for additive and dominance effects, respectively, and* A* (the first homozygote),* H* (heterozygote), and *B* (the second homozygote) indicate the three genotypes. Z_k_ # W_k_ represents element-wise vector multiplication.

The original kinship matrix were normalized by dividing the mean of all diagonal elements of the original matrix so that the diagonal elements are approximately equal to 1. Using the normalized kinship matrix will result in the estimated genetic variance having the same scale as the residual variance.

The *makeH()* function combines information of pedigree and genotypes to construct kinship matrix **H** for both genotyped and ungenotyped individuals used in single-step genomic best linear unbiased prediction (ssGBLUP) method [3, 22]. The formula for **H** matrix is:

6 $$\begin{array}{c}H=\left[\begin{array}{cc}{\mathbf{H}}_{11}& {\mathbf{H}}_{12}\\ {\mathbf{H}}_{21}& {\mathbf{H}}_{22}\end{array}\right]=\left[\begin{array}{cc}{\mathbf{A}}_{11}+{\mathbf{A}}_{12}{\mathbf{A}}_{22}^{-1}\left({\mathbf{G}}_{\mathbf{w}}-{\mathbf{A}}_{22}\right){\mathbf{A}}_{22}^{-1}{\mathbf{A}}_{21}& {\mathbf{A}}_{12}{\mathbf{A}}_{22}^{-1}{\mathbf{G}}_{\mathbf{w}}\\ {\mathbf{G}}_{\mathbf{w}}{\mathbf{A}}_{22}^{-1}{\mathbf{A}}_{21}& {\mathbf{G}}_{\mathbf{w}}\end{array}\right]\end{array}$$

where subscript 1 represent the individuals without genotypes and subscript 2 represent the individuals with genotypes. ${\mathbf{A}}_{11}$, ${\mathbf{A}}_{12}$, ${\mathbf{A}}_{21}$ and ${\mathbf{A}}_{22}$ are constructed by pedigree information.${\mathbf{G}}_{\mathbf{w}}$ is calculated by ${\mathbf{G}}_{\mathbf{w}}=\left(1-w\right){\mathbf{G}}^{\mathbf{*}}+w{\mathbf{A}}_{22}$. The parameter *w* is used adjust the relative weights of the **G** matrix and the **A** matrix. ${\mathbf{G}}^{\mathbf{*}}=a+b\mathbf{G}$, a and b are achieved by:

$$Avg\left(diag\left(\mathbf{G}\right)\right)\times b+a=Avg\left(diag\left({\mathbf{A}}_{22}\right)\right)$$

7 $$\begin{array}{c}Avg\left(offdiag\left(\mathbf{G}\right)\right)\times b+a=Avg\left(offdiag\left({\mathbf{A}}_{22}\right)\right)\end{array}$$

where ${\mathbf{A}}_{22}$ is a submatrix of **A** related to the genotyped individuals; **G** is a additive genomic relationship matrix of genotyped individuals. $\mathrm{A}vg\left(diag\left(\mathbf{G}\right)\right)$ is the average of the diagonal of the **G** matrix. And $Avg\left(offdiag\left(\mathbf{G}\right)\right)$ is the average of the off-diagonal of the **G** matrix.

### Construction of kinship matrix based on microbiome and transcriptome data

The host associated microbiome is known to influence many traits. A number of studies have reported that combining microbiome and genomic information could improve the prediction accuracy compared with only genomic data [23]. The *makeM()* function can easily normalize operational taxonomic units (OTU) as well as calculate the kinship matrix based on microbiome data. The formula for **M** matrix is as follows:

8 $$\begin{array}{c}M=\frac{\mathbf{O}{\mathbf{O}}^{\mathbf{T}}}{n}\#\end{array}$$

where *n* is the number of OUT in population. **O** is the original OTU matrix after natural logarithmic variation and normalization. And for each OUT *j* of individual *i,* the transformation formula is:

9 $$\begin{array}{c}{O}_{ij}=\frac{\mathit{log}\left({X}_{ij}\right)-{\overline{\mathit{log }\left({X}_{ij}\right)}}_{j}}{{sd\left(\mathit{log}\left({X}_{ij}\right)\right)}_{j}}\#\end{array}$$

where ${\mathrm{X}}_{\mathrm{ij}}$ is the abundance of the *j*th OTU of the *i*th individual. ${\mathrm{sd}\left(\mathrm{log}\left({\mathrm{X}}_{\mathrm{ij}}\right)\right)}_{\mathrm{j}}$ is the standard deviation of *j*th OUT in all individuals.

In addition, modeling transcriptome data as predictors in genomic prediction is expected to explain more nonlinear variation or complex biological regulatory processes and has the potential to improve the accuracy of prediction [24]. The *makeT()* function in PyAGH can simply calculate the kinship matrix based on transcriptome data. The formula for **T** matrix is as follows:

10 $$\begin{array}{c}T=R{\mathbf{R}}^{\mathbf{T}}\#\end{array}$$

**R** is the normalized gene expression matrix, and the normalization formula is:

11 $$\begin{array}{c}{r}_{ij}=\frac{{X}_{ij}-\overline{{X }_{j}}}{{sd}_{j}}\#\end{array}$$

where ${X}_{ij}$ is the expression of gene* j* in individual *i*, $\overline{{X }_{j}}$ is the mean of the expression of gene *j* in all individuals, and ${sd}_{j}$ is the standard deviation of the expression of gene *j* in all individuals.

### Pedigree and composition analysis and visualization

Pedigree provides important information for estimating breeding values in the field of plant and animal breeding. To make it easier to use such information, PyAGH provides targeted tools for specific demands, such as detecting common pedigree errors (like offspring born before its parents, individual with two genders, same offspring have different parents, and etc.), selection target individuals (a subset of the whole pedigree), sorting pedigree by birthdate, pedigree visualization, calculating inbreeding coefficients and ancestry coefficients. In addition, principal component analysis (PCA), heatmap and cluster analysis functions were involved in PyAGH to reveal population structure conveniently.

## Results

To support the robustness and speed of the package, we tested the performance of main functions with different cases data in a Linux machine with Intel(R) Xeon(R) Gold 5218 CPU @ 2.30 GHz and 256 GB RAM. First, we compared the makeA function with the Nadiv (https://github.com/matthewwolak/nadiv) package using a dataset containing 100,000 pedigree records. Nadiv is a widely used R package for processing pedigree data. The results of the comparison between the two softwares were shown in Table 3. When the number of records is small, the computational speed of PyAGH and Nadiv is not much different, and even Nadiv is slightly faster than PyAGH. But PyAGH can support a larger number of pedigree data. For example, when the number of records reache 100,000, Nadiv was unable to perform the calculation, while PyAGH took only about 13 min to complete the calculation. This indicates that PyAGH can support a larger amount of pedigree data while maintaining speed compared to Nadiv package when calculating the pedigree additive kinship matrix. In addition, because the first step in calculating the dominance effect kinship matrix based on pedigree data is to calculate the additive effect kinship matrix, i.e., the makeA function is the basis of the makeD function, PyAGH can also support a larger amount of pedigree data for the calculation of the dominance effect kinship matrix.

**Table 3:** Runtime and RAM of construction kinship matrix based on pedigree in PyAGH and Nadiv

Number of records	PyAGH	Nadiv
10,000	3 s (0.3 GB)	2 s (0.3 GB)
20,000	32 s (1.5 GB)	23 s (1.6 GB)
50,000	2min8s (11.9 GB)	1min47s (16.4 GB)
100,000	13min35s (100.6 GB)	–

The numbers of pedigree records verify from 10,000 to 100,000

– means that it cannot be calculated

Next, we tested functions that perform calculations based on genomic data. We compared the makeG function with GCTA software using a dataset containing 10,000 individuals and 1 million SNPs for one chromosome. The runing time for the two software to calculate the additive genomic kinship matrix for different number of individuals are shown in Table 4. Regardless of the number of individuals, PyAGH computed the G matrix faster than GCTA. In addition, PyAGH provides two additional methods for calculating additive kinship matrices in combined populations, whereas GCTA does not calculate. Therefore, using PyAGH makes it easier and faster to perform matrix calculations based on research needs.

**Table 4:** Running time of constructing kinship matrices based on genotypic data in PyAGH and GCTA

Number of individuals	PyAGH	GCTA
1000	10 s	2min32s
3000	51 s	2min36s
5000	1min39s	3min44s
10,000	4min50s	7min40s

Both PyAGH and GCTA used all 64 threads of the machine for computation

We fixing the number of snps at 1,000,000 while varying the numbers of individuals from 1,000 to 10,000

The function makeG_inter in PyAGH, which calculates the dominance effect kinship matrix based on genomic data, was compared with PEPIS platform [25]. PEPIS is a pipeline for estimating epistatic effects in quantitative trait locus mapping and genome-wide association studies. Since PEPIS is a cloud-based platform, we used the test data provided by PEPIS including 1000 individuals and 40,000 SNP for PyAGH testing. Table 5 shows the running time for PyAGH and PEPIS to calculate the kinship matrices of the four dominance effects aa, dd, ad, da, from which it can be seen that the advantage of PyAGH over PEPIS increases as the number of loci increases. At 40,000 loci, the computational speed of PyAGH was about 3 ~ 4 times faster than that of PEPIS. Whether using pedigree or genomic information, PyAGH has speed advantages over other softwares and can support larger data sizes.

**Table 5:** Running time of construction kinship matrix for epistatic effect in PyAGH and PEPIS

Number of SNPs	PyAGH	PEPIS
4000	4 min	5 min
10,000	16 min	34 min
20,000	48 min	121 min
40,000	140 min	488 min

The test data is simulation data used in PEPIS (http://bioinfo.noble.org/PolyGenic_QTL/). We fixing the number of individuals at 1,000 while varying the numbers of SNPs from 4,000 to 40,000

**Fig. 1:**
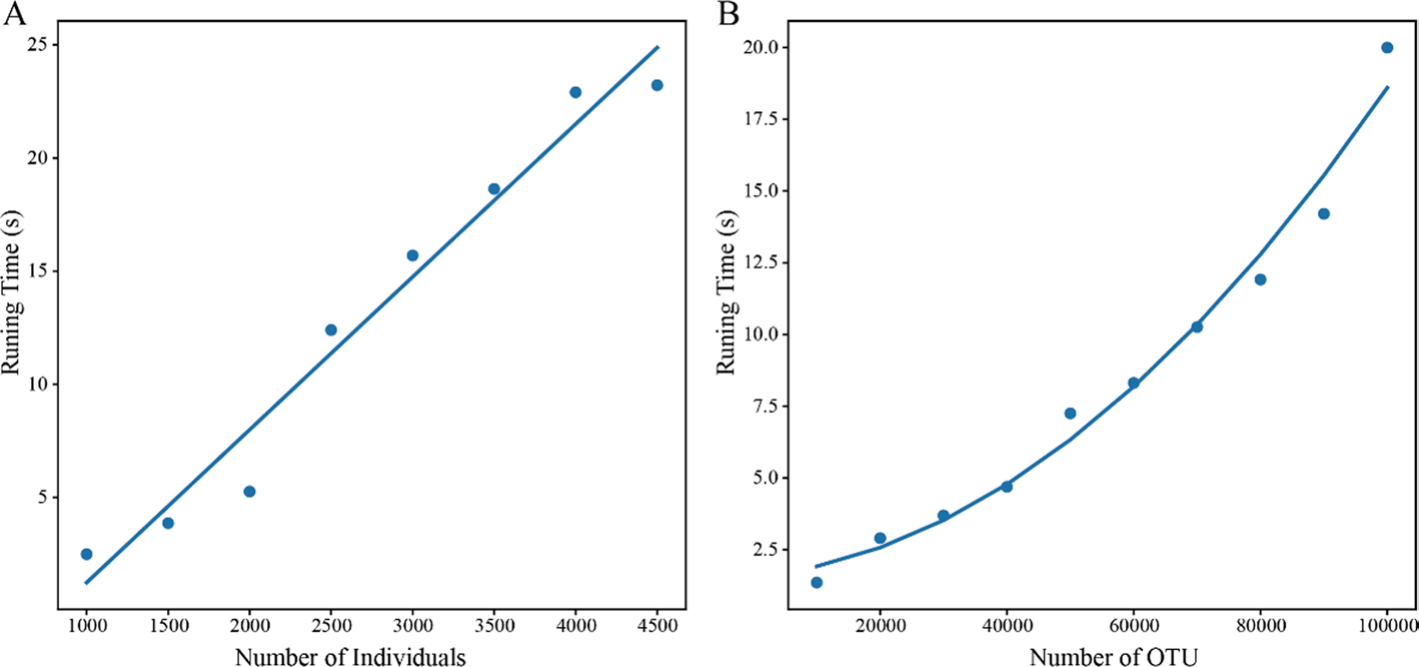
Running time of PyAGH function makeM in different data. **A** fix the number of OTU at 100,000 while varying the numbers of individuals from 1,000 to 4,500. **B** fix the number of individuals at 4,500 while varying the numbers of OTU from 10,000 to 100,000

Because there is no software to calculate the kinship matrix based on microbiome data, we tested PyAGH in a dataset containing 16 s RNA sequencing data of 4500 pigs [26]. The results show that the package can quickly calculate the **M** matrix in the case of meeting the data size of a conventional study. When we fix the number of OTU at 100,000 and the number of individuals varies from 1,000 to 4,500, the time taken increases linearly (Fig. 1A). When we fix the number of individuals at 4,500 while varying the numbers of OTU from 10,000 to 100,000, the time taken increases as a quadratic function (Fig. 1B). For all 4,500 individuals and 100,000 OTU, PyAGH took about 20 s, and it can be seen that our software can quickly normalize the OUT matirx and calculate the kinship matrix.

**Fig. 2:**
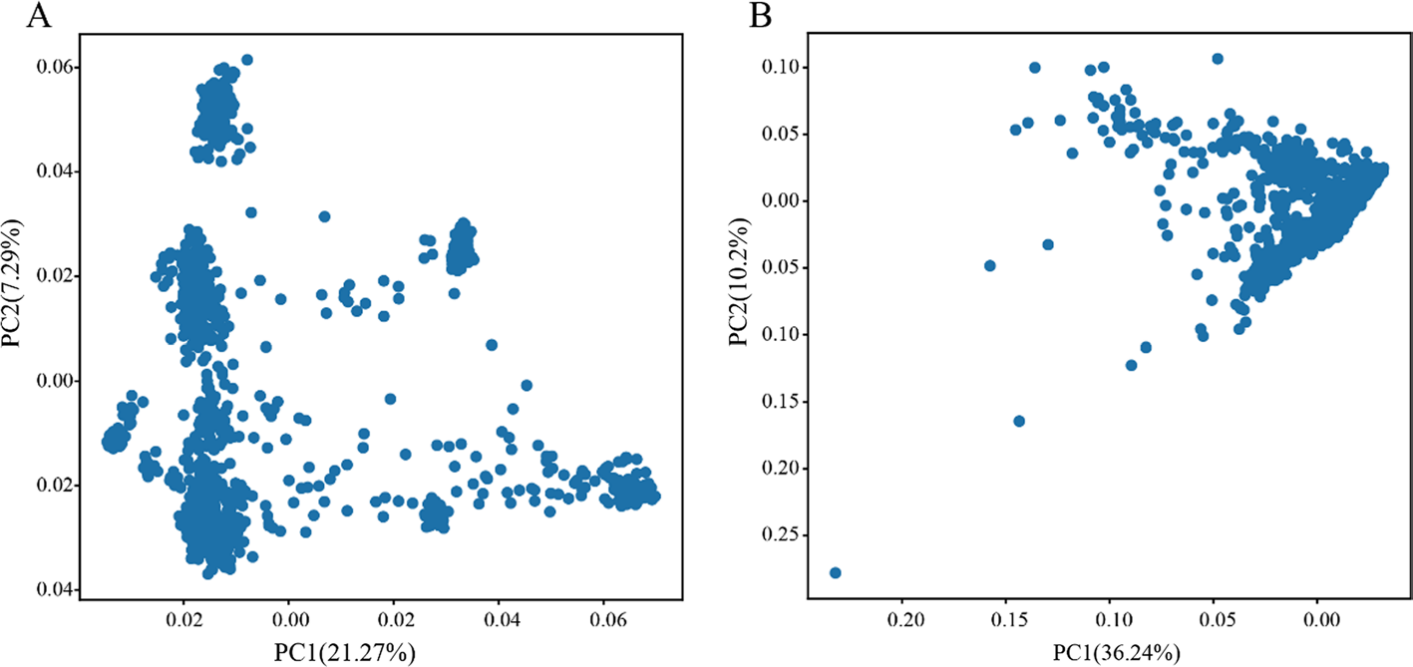
Scatter plots of the first and the second principal components from kinship matrices based on genome (**A**) and transcriptome (**B**), respectively

Gene expression data can provide additional information in genomic prediction and can also be used to further explore the genetic mechanisms of traits in association studies. With the increase of transcriptome sequencing data, the application of transcriptome data in GP and GWAS will increase. PyAGH can quickly and easily calculate kinship matrix based on gene expression abundance data. And we used the gene expression data in muscle tissue of 1321 pigs from FarmGTEx (https://www.farmgtex.org/) as an example [27]. We performed PCA of the kinship matrices based on genomic data (Fig. 2A) and transcriptomic data (Fig. 2B), respectively. The results show that the kinship matrices calculated based on the two data were different, indicating that the transcriptome data provide additional information different from the genome.

**Fig. 3:**
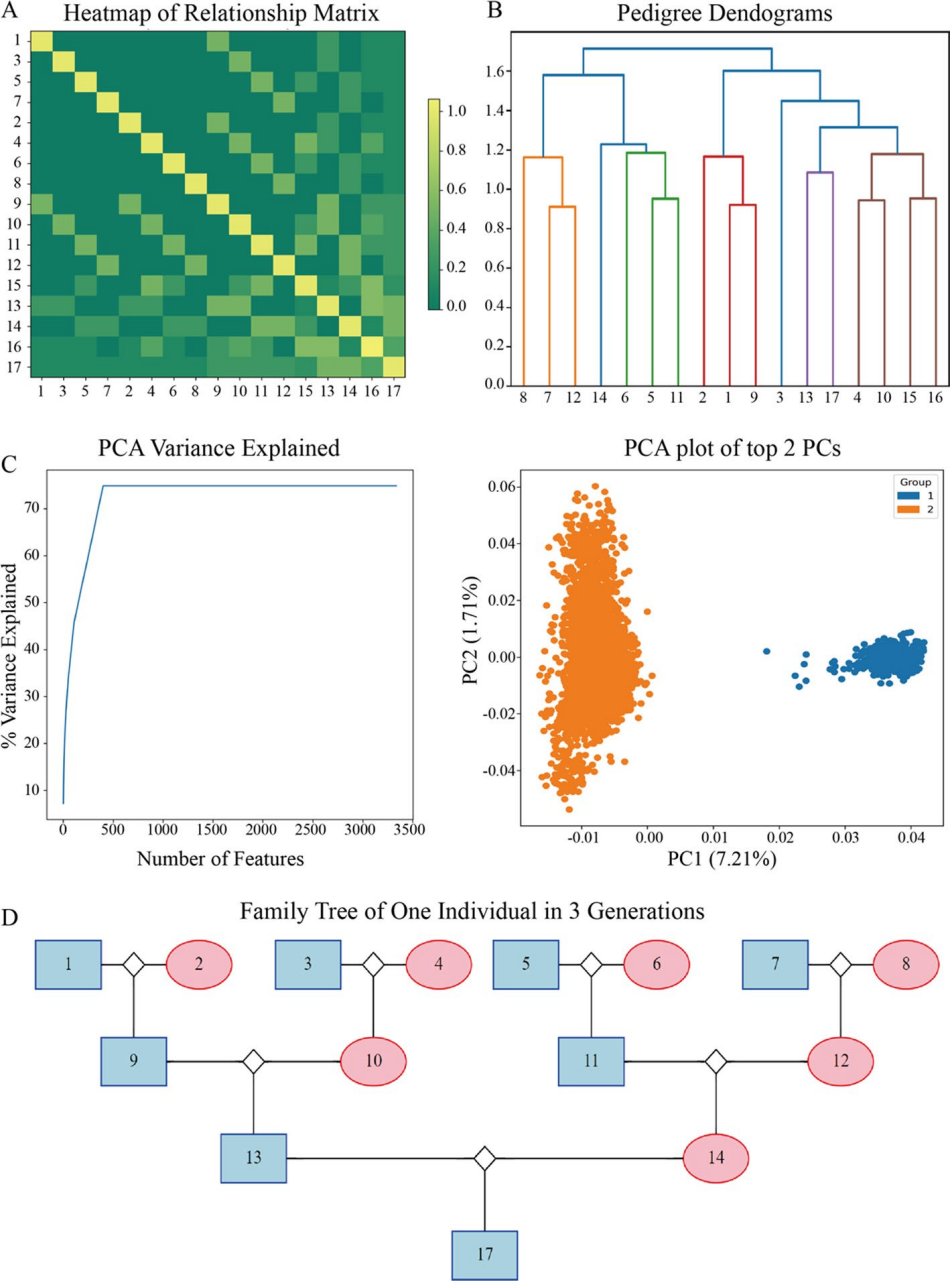
**A** Heat-map of a kinship matrix. **B** The pedigree dendograms of the cluster results. **C** PCA analysis results. The left figure is PCA variance explained based on custom PCA. The right figure is PCA plot of top 2 PCs. **D** Family tree of one individual in three generations

In addition to calculating a variety of kinship matrices, PyAGH can also quickly check pedigree data, extract specific subsets of individuals on demand, and calculate ancestry coefficients and inbreeding coefficients. These features allow the user to easily organize the pedigree data to focus on the next analysis process. At the same time, PyAGH allows for a variety of visualizations including PCA, Heatmap, clustering and family trees. Figure 3A, B shows the heat map and clustering diagram drawn using the example data in the package. Figure 3C shows the results of PCA analysis of the genomic data of two populations using PyAGH. Data were obtained from previous study of two large white pig populations [28]. The left figure is PCA variance explained based on custom PCA. The right figure is PCA plot of top 2 PCs. Figure 3D was a family tree of one specific individual in three generations. This function is useful in production practice.

## Conclusions

In this study, we have presented PyAGH, which is a robust and fast Python package for calculating kinship matrices using pedigree, genotype, microbiome and transcriptome data as well as processing, analyzing and visualizing data and results. This package provides various methods for kinship matrices construction based on additive, dominant and epistatic effects in a single population or combined populations. The PyAGH package has been intensively tested to guarantee the computation correctness and speed. Compared to existing tools, PyAGH exhibited the best performance for constructing a variety of matrices. And the calculation results can be easily used in other softwares, making the process of genome prediction and association studies more convenient. PyAGH is a python package that completes the process of using python for bioinformatics analysis. In the future work, we plan to apply more comprehensive kinship matrix calculation methods and multi-omics data processing to the coming version of PyAGH. In conclusion, PyAGH simplifies the procedure of calculating kinship matrices that are important for prediction or association studies.

## Availability and requirements

Project name: PyAGH.

Project homepage: https://github.com/zhaow-01/PyAGH

Operating System(s): Mac Os, Linux, Windows.

Programming language: Python, C +  + .

Other requirements: All dependencies are handled during the installation.

License: MIT.

Any restrictions to use by non-academic: PyAGH has no restriction.

## Data Availability

The pedigree data underlying this article are available at https://github.com/zhaow-01/PyAGH/tree/main/PyAGH/data. The two pig populations genomic data are available in Alphaindex platform at http://alphaindex.zju.edu.cn/ALPHADB/download.html. The 16 s RNA sequencing data are available in GSA at https://ngdc.cncb.ac.cn/gsa/ database, and can be accessed with accession numbers: CRA006230, CRA006239, CRA006240, CRA006216. The transcriptome data are available in FarmGTEx at http://piggtex.farmgtex.org/. The source code of PyAGH is deposited in a Github repository https://github.com/zhaow-01/PyAGH/tree/main/PyAGH.

## Abbreviations

BLUP: Best linear unbiased prediction
GWAS: Genome wide association studies
GP: Genomic prediction
GBLUP: Genomic best linear unbiased prediction
MAF: Minor alle frequencies
ssGBLUP: Single-step genomic best linear unbiased prediction
OTU: Operational taxonomic units
PCA: Principal component analysis
